# *Vibrio cholerae* as a predator: lessons from evolutionary principles

**DOI:** 10.3389/fmicb.2013.00384

**Published:** 2013-12-10

**Authors:** Stefan Pukatzki, Daniele Provenzano

**Affiliations:** ^1^Department of Medical Microbiology and Immunology, University of AlbertaEdmonton, AB, Canada; ^2^Department of Biomedical Sciences, University of Texas BrownsvilleBrownsville, TX, USA; ^3^Department of Biological Sciences, University of Texas BrownsvilleBrownsville, TX, USA

**Keywords:** *Vibrio cholerae*, intraguild predation, cholera, nutrient acquisition, type VI secretion system, microbiome modification

## Abstract

Diarrheal diseases are the second-most common cause of death among children under the age of five worldwide. Cholera alone, caused by the marine bacterium *Vibrio cholerae*, is responsible for several million cases and over 120,000 deaths annually. When contaminated water is ingested, *V. cholerae* passes through the gastric acid barrier, penetrates the mucin layer of the small intestine, and adheres to the underlying epithelial lining. *V. cholerae* multiplies rapidly, secretes cholera toxin, and exits the human host in vast numbers during diarrheal purges. How *V. cholerae* rapidly reaches such high numbers during each purge is not clearly understood. We propose that *V. cholerae* employs its bactericidal type VI secretion system to engage in intraspecies and intraguild predation for nutrient acquisition to support rapid growth and multiplication.

*Vibrio cholerae* – the causative agent of the diarrheal disease cholera – is an ancient companion of human civilization. Reports of cholera symptoms date back to ancient Greek civilization and Sanskrit writings (Barua, 1992). Records of the disease describing seven cholera pandemics have been maintained since the 19th century (Blake, 1994). Although the discovery is often attributed to Koch (1884), the Italian anatomist Filippo Pacini first identified *V. cholerae* in 1854 as the causative agent of cholera (Bentivoglio and Pacini, 1995). Advances in molecular biology during the late 20th century greatly improved our understanding of *V. cholerae* pathogenesis. The discovery of the ToxR regulon (Miller and Mekalanos, 1984) and characterization of the principal virulence factors cholera toxin (CT) and toxin co-regulated pilus (TCP) shed light on molecular mechanisms that mediate pandemic cholera spread (Herrington et al., 1988). TCP biosynthesis genes are encoded within a horizontally transferred mobile genetic element known as Virulence Pathogenicity Island I (VPI-I) that in addition to TCP and other accessory genes encodes several transcriptional activators and virulence factors required for pandemic spread of *V. cholerae* (Karaolis et al., 1998). TCP functions as a receptor for the CTX-φ filamentous bacteriophage that transduces CT genes into the chromosomes of pandemic *V. cholerae* strains. The contribution of CTX-φ to bacterial pathogenesis brought to light a remarkable example of mutualism between a bacteriophage and a bacterial pathogen, because only *V. cholerae* strains encoding CT are capable of pandemic spread (Waldor and Mekalanos, 1996).

Cholera toxin and additional diarrheagenic factors instruct the epithelial lining of the gut to secrete electrolytes and water that constitute the diarrhea characteristic of cholera (Sharp and Hynie, 1971). However, what nutrient source permits *V. cholerae* to reach bacterial counts reaching up to 10^9^ CFUs/mL during acute cholera is unclear. Digested food is likely not a main source of nutrients for the bacteria, because cholera patients frequently empty their stomachs through vomiting (Chatterjee, 1953) and disease symptoms are often more severe in patients suffering from malnutrition (Dutta et al., 1991). A probable source of energy for *V. cholerae*, especially during the early phases of colonization, is the mucus layer that coats the entire length of the human gastrointestinal tract. In support of this hypothesis, *V. cholerae* secretes the metallo-protease TagA to specifically cleave mucin glycoproteins and select cell-surface glycans (Szabady et al., 2011). The ToxR regulon induces expression of VPI-encoded *tagA* *in vivo* (Bina et al., 2003; Withey and Dirita, 2005). Sialic acids of mucous membranes are another likely nutrient source for *V. cholerae*. The Virulence Pathogenicity Island 2 (VPI-2), a horizontally acquired DNA element, contains several genes for sialic acid transport and catabolism (Chowdhury et al., 2012). Furthermore, HA-protease, a broad-spectrum protease with mucinase, neuraminidase, and additional enzymatic activities, is important for *V. cholerae *dissemination (Häse and Finkelstein, 1991; Finkelstein et al., 1992). Taken together, these findings support the hypothesis that saccharides of mucin and the glycocalyx on the surface of epithelial cells provide carbon and nitrogen as an energy source for *V. cholerae* growth and multiplication during early stages of infection. Thus, mucin-supported growth may be important particularly in the small intestine – the host environment colonized by *V. cholerae*. However, whether host mucins are sufficient to sustain a rapidly multiplying biomass of *V. cholerae* that quickly replenishes between rice-water purges consisting in part of bacteria associated with mucus plugs (Nelson et al., 2007) remains to be demonstrated. We propose that *V. cholerae* engages in predatory behavior to supplement a mucin diet with nutrients acquired from lysed bacteria.

In the environment, *V. cholerae* extracts energy from chitin through what has been collectively coined as the chitin utilization program (Meibom et al., 2004). Remarkably, chitin induces cell density-dependent natural competence in *V. cholerae*. Cholera bacteria switch from degrading exogenous nucleic acids for acquisition into their own pool of nucleotides at low cell density to taking up intact DNA at higher cell densities leading to transformation (Blokesch and Schoolnik, 2008). Chitin therefore plays a critical role in the environmental life style of *V. cholerae* as a source of nutrients and as a signal for uptake of either nucleotides or whole DNA sequences. *V. cholerae*’s ability to bind chitin is mediated by a secreted *N*-acetylglucosamine binding protein (GbpA) that also functions as the adhesin for attachment to mucin in the host intestines (Kirn et al., 2005; Wong et al., 2012). Considering the overlapping roles of mucin and chitin in *V. cholerae* adherence, colonization and nutrient acquisition, this analogy appears to become an inescapable theme. Perhaps *V. cholerae’s* ancestral role as a chitin degrader in marine environments is the reason for the ability of the cholera bacterium to bind mucin in the human intestine.

Chitin binding likely preceded cholera bacteria’s ability to bind mucin in the small intestine because all strains are capable of persisting in the environment, but not all *V. cholerae* are host-colonization competent. Analogously, bacterial predation likely evolved before *V. cholerae* became intestinal colonizers because single cells competed against each other in environmental reservoirs prior to emergence of multicellularity. Therefore, acquisition of DNA coding instructions for the biosynthesis and delivery of bactericidal effector molecules followed by natural selection may have provided predatory cells a formidable competitive advantage in any given niche. However, effective predation requires mechanisms to distinguish prey from kin as is the case for *Escherichia coli*, a bacterial species adapted to reside in vertebrate intestines. Coligenic *Escherichia coli* secrete episomally encoded bactericidal proteins called colicins (Jacob et al., 1952) that kill related bacteria, but exclude kin bacteria protected by means of cognate immunity proteins (Chak and James, 1984). Lysed neighboring prey, which may include non-coligenic *Escherichia coli* as well as coligenic bacteria expressing different immunity proteins, can become a nutrient source for predatory colicin-producing *Escherichia coli* (Leisner and Haaber, 2012). However, the best characterized examples of nutrient acquisition as a direct result of predation are found among the deltaproteobacteria: *Bdellovibrionaceae* are diminutive aquatic dwellers that penetrate the periplasm of larger Gram-negative prey where they utilize macromolecules as a source of nutrients and divide until they exit the host to repeat the cycle (Stolp and Starr, 1963). Another example is Myxobacteria found in most terrestrial soils and aquatic environments that subsist from nutrients acquired from both bacterial and fungal prey. In contrast to *Bdellovibrionaceae*, Myxobacteria display cooperative behavior through group swarming and predation (Berleman and Kirby, 2009) which culminates in the biosynthesis of antibiotics and enzymes (Rosenberg et al., 1977) that kill and convert a wide range of prey cells into growth substrates utilized by the predators for growth and multiplication (Morgan et al., 2010). Perhaps not coincidentally *Myxococcus xanthus* was found to harbor an intact type VI secretion system (T6SS; Bingle et al., 2008).

Interactions between different species can broadly be divided into the following categories: neutralism, competition, predation/parasitism, mutualism, commensalism, and amensalism. Intraguild predation is a combination of competition and predation that occurs when neighboring species competing for limited resources within the same niche assume predatory behavior toward each other to acquire nutrients (Polis et al., 1989). Intraguild predation differs from classical predation because it reduces competition and also differs from traditional competition because the predator directly benefits energetically from the demise of organisms that compete for the same resource pool (Polis et al., 1989). The potential benefits of intraguild predation in intestinal colonization are evidenced by the observation that several bacterial species (Cheng et al., 1973) including *Escherichia coli* (Holme and Cedergren, 1961) and *V. cholerae* (Bourassa and Camilli, 2009) store glycogen in cytoplasmic inclusions during their tenure in the host. In addition to removing competitors from the colonization niche, predation would provide immediate energetic benefits from lysed bacteria to predators like *V. cholerae* upon entry into and before exiting the human host.

*Vibrio cholerae* likely engages in predatory behavior in the marine environment where it spends considerable time of its lifecycle. This would require a genetically conserved, ancestral bactericidal mechanism capable of efficiently discriminating kin cells from prey bacteria. Yet, *V. cholerae* is a highly diverse species as each genome sequenced appears to consist of a patchwork of horizontally acquired genetic elements. Any given *V. cholerae* strain harbors approximately 3,700 genes, while the *V. cholerae* pan-genome consists of ~6,500 genes (Vesth et al., 2010). In spite of the occasional encounter and explosive population expansion in human hosts, *V. cholerae* is primarily a marine inhabitant; its genetic diversity may reflect strain-specific adaptation to specific niches. All pandemic strains harbor CTX-φ and VPI-I (Dziejman et al., 2002); however, several non-pandemic strains are also capable of causing cholera. For example, some environmental strains have been found to code a type III secretion system (T3SS) shown to be a potent diarrheagenic factor in the infant mouse model (Dziejman et al., 2005). Regardless, all strains sequenced thus far harbor the genes for a highly conserved T6SS. The presence of T6SS genes in ~25% of all sequenced Proteobacteria (Bingle et al., 2008) suggests an ancestral origin of the secretion system that may have preceded the evolutionary divergence of *Vibrio* lineages.

To engage in predation (MacIntyre et al., 2010; Unterweger et al., 2012), *V. cholerae* employs a T6SS consisting of a polymeric protein nanotube bearing remarkable structural and functional similarity to the bacteriophage tail complex (Leiman et al., 2009). This highly dynamic tube is rapidly assembled in the cytosol and acts as a scaffold for an outer sheath that upon contraction ejects the inner tube and delivers a puncturing device on the tip into adjacent cells (Basler and Mekalanos, 2012). The discovery of T6SS in* V. cholerae* was originally linked to pathogenesis because an effector that crosslinks actin causes toxicity in *Dictyostelium discoideum* (Pukatzki et al., 2007). Recently, three T6SS effectors with homology to lipases (Dong et al., 2013), pore-forming proteins (Miyata et al., 2013), and peptidoglycan-degrading enzymes (Brooks et al., 2013; Dong et al., 2013) were found to exclusively target bacteria. Only a fraction of *V. cholerae* strains examined to date express the T6SS under laboratory conditions; yet, the secretion system has been linked to intestinal inflammation in the infant mouse (Ma and Mekalanos, 2010) and infant rabbit (Zheng et al., 2010) cholera models. Furthermore, an *in vivo* expression technology (IVET) screen employing non-toxigenic *V. cholerae* strains carried out in human volunteers suggested that T6SS genes are transcribed in the human host (Lombardo et al., 2007). These findings are supported by RNA-Seq experiments that show T6SS gene transcription in mouse intestines employing a toxigenic *V. cholerae* strain that does not express the secretion apparatus under laboratory conditions (Mandlik et al., 2011). Collectively these reports provide abundant support for a role of *V. cholerae* T6SS expression *in vivo*.

Evidence that T6SS genes are also likely expressed in the environment emerged from a report showing that salt concentrations and temperatures analogous to those encountered by *V. cholerae* in the marine environment induce T6SS biosynthesis (Ishikawa et al., 2012). These experiments employed toxigenic *V. cholerae *strains that otherwise do not express the secretion apparatus under laboratory conditions. In addition, we recently showed that non-toxigenic *V. cholerae* strains isolated from the Rio Grande Delta express their T6SS constitutively (Unterweger et al., 2012) supporting a role for the secretion system in the marine life style of this species. Perhaps more interestingly, this strain collection allowed us to test the hypothesis that *V. cholerae*’s T6SS immunity system (Dong et al., 2013; Miyata et al., 2013) mediates resistance to strain-specific T6SS-mediated toxicity. In summary, our results showed that the extent of killing between unrelated strains differs leading to speculations that distinct toxin/antitoxin sets are harbored by different *V. cholerae* and that T6SS-mediated selective intraspecies killing permits strains to distinguish self from non-self (Unterweger et al., 2012).

*Vibrio cholerae* does not utilize the T6SS exclusively to kill bacteria of its own species; several additional prokaryotes are susceptible to T6SS-dependent killing. Some of these susceptible bacteria include *V. communis*,* V. harveyi*,* V. mimicus*,* V. fischeri*,**and* Pseudoalteromonas phenolica* isolated from the same environmental niches as *V. cholerae* (Unterweger et al., 2012). Other bacteria susceptible to T6SS-mediated killing are less likely to encounter *V. cholerae* in the marine environment; these include *Escherichia coli*, *Salmonella typhimurium, *and* Citrobacter* *rodentium*. Thus far, only Gram-negative bacteria have demonstrated susceptibility to *V. cholerae* T6SS killing, while *Pseudomonas aeruginosa* was recently shown to be capable of counter attacking cholera bacteria utilizing its own T6SS (Basler et al., 2013). Gram-positive bacteria examined thus far such as *Enterococcus faecalis, Bacillus subtilis, Listeria monocytogenes*,**and *Staphylococcus aureus* are resistant to *V. cholerae* T6SS bactericidal activity (MacIntyre et al., 2010). One possible explanation is that Gram-positive bacteria are naturally refractory to *V. cholerae*’s T6SS’s bactericidal effect; alternatively we cannot rule out the possibility that additional effectors targeting Gram-positive species remain to be discovered and characterized. Either way, susceptibility of the host microbiome to T6SS-mediated killing may be one of numerous factors contributing to *V. cholerae*’s ability to successfully colonize the host.

The host microbiome experiences profound changes during cholera infections. Commensal bacteria belonging to the *Bacteroides*, *Firmicutes,* and *Actinobacteria* phyla are displaced while harmful *Proteobacteria* prosper (Monira et al., 2013). Benign *Escherichia coli* K12 capable of colonizing the human small intestine implanted at high inocula failed to maintain a foothold in the intestines of volunteers suffering from acute cholera (Gorbach et al., 1970). This outcome may be, in part, due to K-12’s high susceptibility to *V. cholerae* T6SS killing (MacIntyre et al., 2010). While shifts in bacterial populations and modification to the host microbiome during cholera infections are likely attributed to their displacement by water and electrolyte efflux from host cells in the intestine as a result of fulminant diarrhea, we propose that T6SS-mediated predation contributes to these shifts. We hypothesize that *V. cholerae* engage their T6SS in intraspecies competition by killing cholera strains harboring different effector/immunity proteins that compete for the same niche in the small intestine upon entry into the host, leading to kin selection and clonal expansion. Subsequently, killing of T6SS-sensitive bacteria competing for the same niche in the large intestine may allow *V. cholerae* to engage in intraguild predation thereby shifting bacterial populations to its advantage and acquire nutrients from prey. For either scenario, we suggest that *V. cholerae*’s T6SS-mediated bacterial killing has contributed to its success as an enteric pathogen.

## Conflict of Interest Statement

The authors declare that the research was conducted in the absence of any commercial or financial relationships that could be construed as a potential conflict of interest.

## AUTHOR CONTRIBUTIONS

Daniele Provenzano and Stefan Pukatzki wrote the manuscript and conceived the ideas therein.
